# Negligible evidence for detrimental effects of *Leucocytozoon* infections among Emperor Geese (*Anser canagicus*) breeding on the Yukon-Kuskokwim Delta, Alaska

**DOI:** 10.1016/j.ijppaw.2021.08.006

**Published:** 2021-08-21

**Authors:** Andrew M. Ramey, Raymond M. Buchheit, Brian D. Uher-Koch, John A. Reed, M. Andreína Pacheco, Ananias A. Escalante, Joel A. Schmutz

**Affiliations:** a US Geological Survey Alaska Science Center, Anchorage, AK, USA; b Department of Ecology, Montana State University, Bozeman, MT, USA; c Biology Department/Institute of Genomics and Evolutionary Medicine (iGEM), Temple University, Philadelphia, PA, USA

**Keywords:** Avian malaria, Haematozoa, Haemosporidian, Incubation, *Leucocytozoon*, Mass, Parasite, Survival

## Abstract

Emperor Geese (*Anser canagicus*) are iconic waterfowl endemic to Alaska and adjacent areas of northeastern Russia that are considered to be near threatened by the International Union for Conservation. This species has been identified as harboring diverse viruses and parasites which have, at times, been associated with disease in other avian taxa. To better assess if disease represents a vulnerability for Emperor Geese breeding on the Yukon-Kuskokwim Delta, Alaska, we evaluated if haemosporidian parasites were associated with decreased mass or survival among adult female nesting birds captured during 2006–2016. Through molecular analyses, we detected genetically diverse *Leucocytozoon*, *Haemoproteus*, and *Plasmodium* parasites in 28%, 1%, and 1% of 607 blood samples screened in triplicate, respectively. Using regression analysis, we found evidence for a small effect of *Leucocytozoon* infection on the mass of incubating adult female Emperor Geese. The estimated mass of infected individuals was approximately 43 g (95% CI: 20–67 g), or approximately 2%, less than uninfected birds when captured during the second half of incubation (days 11–25). We did not, however, find support for an effect of *Leucocytozoon* infection on survival of adult female nesting Emperor Geese using a multi-state hidden Markov framework to analyze mark-resight and recapture data. Using parasite mitochondrial DNA cytochrome *b* sequences, we identified 23 haplotypes among infected Emperor Geese. *Leucocytozoon* haplotypes clustered into three phylogenetically supported clades designated as ‘*L. simondi* clade A’, ‘*L. simondi* clade B’, and ‘other *Leucocytozoon*’. We did not find evidence that parasites assigned to any of these clades were associated with differential mass measures among nesting adult female Emperor Geese. Collectively, our results provide negligible evidence for *Leucocytozoon* parasites as causing detrimental effects to adult female Emperor Geese breeding on the Yukon-Kuskokwim Delta.

## Highlights

• Emperor Geese are consistently infected with *Leucocytozoon* parasites.
• We found evidence for a small effect of infection on mass of incubating females.
• We did not find support for an effect of *Leucocytozoon* infection on survival.

## Graphical abstract

**Figure:**
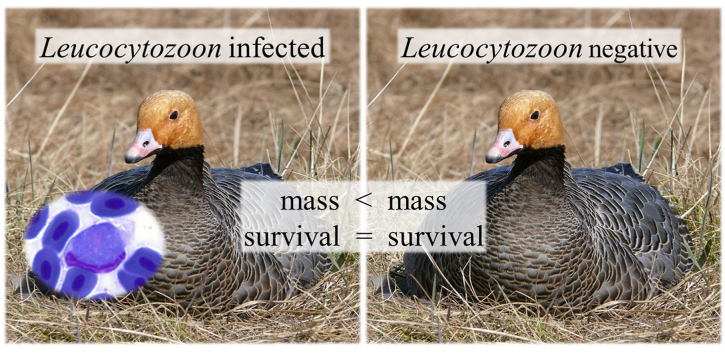

## 1 Introduction

Emperor Geese (*Anser canagicus*) have a geographically limited distribution within coastal western Alaska and northeastern Russia, and adjacent waters (Schmutz et al., 2020). This species is less abundant than other North American geese, with an estimated population during 2007–2014 of approximately 130,000 birds (Pacific Flyway Council 2016). Emperor Geese are a culturally important subsistence resource in western and southwestern Alaska and are subject to limited sport harvest (Pacific Flyway Council 2016). The conservation status of Emperor Geese is considered ‘near threatened’ by the International Union for Conservation given the species' limited geographic range, relatively small population size, and harvest pressure (BirdLife International 2016).

Though not well understood, disease may also represent a potential vulnerability for Emperor Geese. For example, diverse coronaviruses (Muradrasoli et al., 2010), influenza A viruses (Reeves et al. 2013, 2018; Ramey et al., 2019), paramyxoviruses (Ramey et al., 2013), and haemosporidian parasites (Hollmén et al., 1998; Ramey et al., 2014; Buchheit et al., 2021a) have previously been identified in Emperor Geese inhabiting Alaska and Russia. Various strains and lineages of these viral and parasitic agents have previously been associated with disease in wild and domestic birds (Valkiunas 2004; Swayne 2009; Dimitrov et al., 2016; Lee et al., 2017, Miłek and Blicharz-Domańska, 2018). Though none of these infectious agents has been associated with clinical disease in Emperor Geese, potential detrimental subclinical effects of infection have not yet been evaluated in these birds.

In this study, we sought to assess if haemosporidian parasites may be associated with decreased mass or survival among breeding adult female Emperor Geese, suggestive of detrimental effects of infection on host fitness and sub-clinical disease. Specifically, we aimed to: (1) estimate the detection probability and prevalence of haemosporidian parasites among Emperor Geese captured on the Yukon-Kuskokwim Delta during 2006–2016; (2) assess potential impacts of haemosporidian infection on the mass of adult female Emperor Geese during incubation as well as the inter-annual survival of infected birds of this same age/sex cohort; and (3) explore the genetic diversity of haemosporidians infecting Emperor Geese relative to potential associations with fitness costs of infection. We conducted our investigation based upon previous reports for associations between haemosporidian infection in waterfowl and possible detrimental fitness effects including anemia (Desser and Ryckman 1976), reduced body condition (Shutler et al., 1999; Meixell et al., 2016; Fleskes et al., 2017, Merrill et al., 2018), and decreased survival (Herman et al., 1975; Sorci and Møller 1997).

## 2 Methods

### 2.1 Study area and sampling

Emperor Geese were captured at breeding sites adjacent to the Manokinak River (approx. 61.2° N, 165.1° W) on the Yukon-Kuskokwim Delta of Alaska during nest incubation in May and June 2006–2016 and during brood-rearing in August 2014. Incubating females were caught on nests utilizing bow or mist nets, and molting flightless adults and unflighted goslings were herded into corrals and sampled during brood drives. Capture and sampling procedures were conducted under U.S. Geological Survey (USGS) federal bird banding permit #20022 and reviewed, as applicable, by the Animal Care and Use Committee at the USGS Alaska Science Center (2008–17, 2016–03). Whole blood samples were collected from captured individuals using jugular venipuncture and stored in Longmire buffer (Longmire et al., 1988). Captured geese were weighed using hanging scales or electronic balances and differentiated by sex through cloacal examination. The nest initiation date for birds captured at nest sites was estimated via egg floating and/or candling (Fischer et al., 2017).

### 2.2 DNA extraction and molecular identification of haemosporidian parasites

Methodology for molecular assessment of haemosporidian infection generally followed Ramey et al. (2012) with few exceptions as indicated in the brief description that follows. DNA was extracted from blood samples using the DNeasy Blood and Tissue Kit (Qiagen, Valencia, USA) per the manufacturer's protocol. Extracted DNA was screened in triplicate to assess the presence of haemosporidians using a nested PCR as described by Hellgren et al. (2004). A minimum of one negative control was incorporated every 24 wells in each set of PCR reactions. Amplified products were visualized on 0.8% agarose gels stained with Gel Red Nucleic Acid Gel Stain (Biotium, Hayward, USA). Products appearing to correspond to our 479 base pair (bp) mitochondrial DNA (mtDNA) cytochrome *b* (*cyt b*) target were treated with ExoSap-IT (USB Inc., Cleveland, USA) and sequenced with identical primers used for PCR with BigDye Terminator version 3.1 mix (Applied Biosystems, Foster City, USA) on an Applied Biosystems 3730xl automated DNA sequencer (Applied Biosystems, Foster City, USA). Sequence data were cleaned and edited using Sequencher version 5.1 (Gene Codes Corp., Ann Arbor, USA).

Parasite infections were characterized to genus (*Plasmodium*, *Haemoproteus*, or *Leucocytozoon*) using the nucleotide BLAST function (Altschul et al., 1997) available through the National Center for Biotechnology Information (NCBI). Assignment of sequences to parasite genus was based on the top NCBI BLAST result with a minimum max identity score of 90%. A sample was considered positive for a parasite infection if any one of the triplicate PCR reactions resulted in a bi-directional double-stranded mtDNA *cyt b* target product that was verified through genetic sequencing. Samples from which only single stranded or otherwise ambiguous molecular products were obtained were considered haemosporidian negative. Samples were classified as co-infected with parasites of more than one genetic lineage if the sequenced and cleaned target product for one or more nested PCR runs yielded > one ambiguous nucleotide or if > one mtDNA *cyt b* haplotype was inferred through resolution with SeaView v4.3.5 (Gouy et al., 2010). Only sequences with ≤ one ambiguous nucleotide within a mtDNA *cyt b* haplotype were included in downstream genetic analyses.

To confirm the presence of quality DNA within extracted samples, a 481 bp fragment of the mtDNA cytochrome oxidase I (*COI*) gene of the Emperor Goose host was amplified using 2 μl of template DNA, primers specifically designed for geese (Ramey et al., 2014), and the same proportion of reagents as previously reported for the nested PCR (Hellgren et al., 2004). Thermalcycling conditions followed Kerr et al. (2007).

### 2.3 Occupancy modeling and assessment of detrimental effects of parasite infection

To estimate detection probability (ρ_1_) and prevalence (ψ_1_), we analyzed results from triplicate screening of each DNA extraction for haemosporidians in an occupancy modeling framework. More specifically, we assessed variation in ρ_1_ and ψ_1_ with a parameter for sample year using occupancy modeling procedures in program MARK (version 9.0; White and Burnham 1999). For this analysis, we included information derived from all blood samples (n = 607), including those from hatch-year (n = 52) and male (n = 37; 28 of which were also hatch-year) birds sampled as part of brood drives in 2014. We limited our estimates of detection probability and prevalence to parasites of the genus *Leucocytozoon* given few detections for *Plasmodium* and *Haemoproteus* parasites in our data set (see Results). We used Fletcher's ĉ to check for overdispersion in the data and assess the fit of our most highly parameterized model (ρ_1_ (Year) ψ_1_ (Year)) to the data. Our estimate of Fletcher's ĉ was 2.22, and we adjusted ĉ in MARK accordingly. We assessed model performance using quasi-Akaike Information Criterion adjusted for small sample sizes (QAICc).

We next performed a regression analysis to assess the potential impacts of haemosporidian infection on the mass of female Emperor Geese during incubation. Specifically, we assessed whether *Leucocytozoon* parasite infections (i.e., those comprising >90% of our dataset; see Results) were associated with lower mass during incubation for female Emperor Geese. For this analysis, we constrained our data to mass measures after the 10th day of incubation (when field crews were present), and observations for birds with mass measures ≥1200 g but ≤2100 g (to remove potentially spurious measures). Constraining data in this way resulted in the omission of six mass measures, which we considered outliers relative to our data set and prior reports (Thompson and Raveling 1987; Schmutz et al., 2020), and which may have been influenced by observer error in the field (e.g., failing to tare electronic balances prior to measuring mass). For the regression analysis, we included data on the infection status of 337 individual female geese using triplicate parasite screening results for 493 blood samples. Numerous (n = 113) individuals were captured and bled in multiple years (≤6), and infection status was assessed from each sample. We included mass measures for the same individual from different years in our final data set, relaxing the assumption of independence of measures in modeling efforts, given inter-annual variability in mass. We contrasted six models in our regression analysis to explain variability in the mass of incubating female Emperor Geese incorporating parameters for day of incubation (Inc), *Leucocytozoon* infection status (Leu), and year. We assessed model performance by applying an information-theoretic approach (Burnham and Anderson 2002) and using the Akaike Information Criterion adjusted for small sample size (AICc).

To evaluate the impact of haemosporidian infection on adult female Emperor Goose survival and resighting probabilities, we used mark-resight and recapture data for 424 tagged adult females from the same 11 year interval assessed in regression analysis (2006–2016). Differences in sample size between this survival analysis and the previously described regression evaluating possible effects of infection on mass were on account of the inclusion of numerous unsampled birds of unknown infection status in the survival analysis and the exclusion from the regression analysis of some tagged individuals from which mass data was not obtained. For the survival analysis, we used a multi-state hidden Markov framework (Conn and Cooch 2009) in Program MARK to account for state uncertainty where individually marked Emperor Geese were resighted but no infection status data were available (i.e., resighted alive but not recaptured). Parameters in these models included survival probability (ϕ), resight probability (ρ_2_), state transition probability (ψ_2_), probability of an event at first capture (π), and the probability of accurate state assessment (δ). We used two states in our analyses to account for infection status of individuals (*Leucocytozoon* infected and uninfected), and one event to account for detection of a marked individual where the infection status was uncertain. We evaluated a candidate set of eight models without covariates. We evaluated potential change in survival over time, but small sample sizes prevented us from evaluating temporal variation between parameters and infection states. We used a combination of logit and mlogit link functions to bound parameter estimates. As with the regression analysis, we constrained our data to parasites of the genus *Leucocytozoon* and assessed model performance using AICc.

### 2.4 Genetic and phylogenetic analyses

To identify the genetic diversity of haemosporidians infecting Emperor Geese, and to explore potential associations between parasite genotype and fitness metrics for incubating female birds, we used mtDNA *cyt b* sequences, a marker widely used to investigate haemosporidians in birds (Pacheco et al., 2018). Specifically, we estimated the frequency of distinct haplotypes, compared them to those available on public databases, and created a median-joining minimum spanning network for the haplotypes of the most common parasite genus detected (i.e., *Leucocytozoon*; see Results). To first identify the number of parasite mtDNA *cyt b* haplotypes among our sample, we constructed three alignments, one for each *Plasmodium*, *Haemoproteus*, and *Leucocytozoon* sequence generated from our sample collection. We performed alignments using ClustalX v2.0.12 and Muscle as implemented in SeaView v4.3.5 (Gouy et al., 2010). Alignments for *Plasmodium*, *Haemoproteus*, and *Leucocytozoon* sequences were 393 base pairs (bp), 320 bp, and 290 bp, respectively. Identical sequences within each alignment were considered to represent the same parasite haplotypes. Next, each parasite haplotype was compared to those reported on the MalAvi (Bensch et al., 2009) and GenBank (Benson et al., 2012) public databases (accessed 25–31 March 2021) to identify identical or similar sequences previously reported for haemosporidians infecting wild birds. Subsequently, to depict the relative frequency of the most commonly detected parasites in sample collection and their comparative genetic distances to one another, we estimated a median-joining minimum spanning network for *Leucocytozoon* haplotypes using the software Network, version 10.2.0.0 (Bandelt et al., 1999) and calculated genetic distance using the p-distance method as implemented in MEGA version 7.0.14 (Kumar et al., 2016).

To infer the genetic relationships among the observed haplotypes in our sample and those closely related as identified through our query of MalAvi and Genbank databases, we estimated phylogenetic trees using Bayesian methods as implemented in MrBayes v3.2.6 (Ronquist and Huelsenbeck 2003). For *Plasmodium*, we used genetic information for one representative sequence for each parasite haplotype we detected in Emperor Geese, sequences from well-known *Plasmodium* morphospecies, and information for *L. caulleryi* and *Haemoproteus* spp. (as outgroups) in our 393 bp alignment. For *Haemoproteus*, we analyzed each haplotype detected in Emperor Geese with four previously reported lineages of unknown morphospecies and 16 sequences from previously identified morphospecies that emerged from our query of public databases. Seven sequences of *Leucocytozoon* or *Haemoproteus* spp. were used as an outgroup in our 320 bp alignment. For *Leucocytozoon*, our 320 bp alignment was comprised of one representative sequence for each distinct parasite haplotype we detected in this study, sequences from 15 previously reported lineages of unidentified *Leucocytozoon* species, and information for 13 previously identified *Leucocytozoon* morphospecies. The sequences of *Leucocytozoon caulleryi* and *Haemoproteus* spp. were used as outgroups. In all cases, the phylogenetic trees were estimated using the Bayesian method with the default priors and the best model that fit the data (general time-reversible model with gamma-distributed substitution rates and a proportion of invariant sites, GTR +Γ + I). This model was selected as it had the lowest Bayesian Information Criterion (BIC) score, as estimated by MEGA v7.0.14 (Kumar et al., 2016). The posterior probability was inferred for the nodes by sampling every 1000 generations from two independent chains lasting 3 × 10^6^ Markov Chain Monte Carlo (MCMC) steps. Chains were assumed to have converged once the average S.D. of the posterior probability was <0.01 and the value of the potential scale reduction factor (PSRF) was between 1.00 and 1.02 (Ronquist and Huelsenbeck 2003). Once convergence was reached, 25% of the samples were discarded as burn-in.

### 2.5 Assessment of effects of parasite infection on mass incorporating genetic information

To explore potential associations between genetic lineages of parasites and possible detrimental fitness effects, we related the genetic haplotypes for *Leucocytozoon* infected incubating female Emperor Geese to measures of mass used in our regression analysis *post hoc*. In particular, we sought to identify if groups of phylogenetically related parasite haplotypes were consistently associated with lower mass measures. We characterized *Leucocytozoon* infections among nesting female birds into three groups based upon the haplotypes detected and the results of our phylogenetic analysis: (1) *L. simondi* clade A (including co-infections with more than one *L. simondi* clade A haplotype), (2) *L. simondi* clade B (including co-infections with more than one *L. simondi* clade B haplotype), or (3) other/mixed *Leucocytozoon* (including co-infections with haplotypes not exclusively nested with *L. simondi* clade A or *L. simondi* clade B). We then conducted a linear regression using the program R (version 4.0.5; R Core Team 2021) to compare mass as a function of incubation day among the geese infected with *Leucocytozoo*n parasites assigned to three phylogenetic clades as previously described. Given the lack of support for an association between survival and *Leucocytozoon* infection (see Results), we did not explore associations between survival and specific *Leucocytozoon* clades. Furthermore, we did not assess possible associations between *Haemoproteus* or *Plasmodium* parasite haplotypes and mass or survival given the few detections of parasites of these two genera (see Results).

All data supporting conclusions in this product have been made publicly available in an associated data release (Buchheit et al., 2021b) and via GenBank (accession numbers MW885252–MW885274, MW960777–MW960958, and MW990224).

## 3 Results

Through the screening of 607 blood samples collected from Emperor Geese inhabiting the Yukon-Kuskokwim Delta, Alaska during the 11-year interval from 2006 to 2016, we found consistent evidence for infection with parasites of the genus *Leucocytozoon* among 11–45% of samples collected in each year (Table 1). In contrast, the molecular detection of *Plasmodium* and *Haemoproteus* parasites was more sporadic with the detection of parasites of these genera in samples collected from three and five years, respectively, including no more than 5% of samples collected in any given year (Table 1). The molecular methods used did not provide information regarding the intensity of these haemosporidan infections. Most geese that were sampled in more than one year (82/113 or 73%) remained either negative (n = 62) or positive (n = 20) for haemosporidian parasite infection(s) across years (Buchheit et al., 2021b). However, a smaller proportion of birds (31/113 or 27%) changed status from haemosporidian negative to haemosporidian positive (n = 17), haemosporidian positive to haemosporidian negative (n = 11), or some combination thereof (i.e., changed infection status more than once; n = 3) (Buchheit et al., 2021b).

**Table 1:** Summary of molecular screening of blood samples collected from Emperor Geese captured on the Yukon-Kuskokwim Delta, Alaska during 2006–2016 for haemosporidian parasites.

Year	Breeding stage at sampling	Samples collected and screened	*Leucocytozoon* positive (%)	*Haemoproteus* positive (%)	*Plasmodium* positive (%)
2006	Nesting	28	3 (11%)	0 (0%)	0 (0%)
2007	Nesting	41	6 (15%)	0 (0%)	0 (0%)
2008	Nesting	35	10 (29%)	1 (3%)	1 (3%)
2009	Nesting	47	21 (42%)	0 (0%)	0 (0%)
2010	Nesting	41	9 (22%)	2 (5%)	0 (0%)
2011	Nesting	37	10 (27%)	1 (3%)	0 (0%)
2012	Nesting	40	14 (35%)	0 (0%)	0 (0%)
2013	Nesting	57	18 (32%)	1 (2%)	2 (4%)
2014	Nesting	28	6 (21%)	0 (0%)	0 (0%)
2014	Brood rearing	97	15 (15%)	3 (3%)	1 (1%)
2015	Nesting	99	39 (39%)	0 (0%)	0 (0%)
2016	Nesting	57	18 (32%)	0 (0%)	0 (0%)
all years		607	169 (28%)	8 (1%)	4 (1%)

Through the application of an occupancy modeling approach, we estimated our molecular detection probability for *Leucocytozoon* parasites to be relatively high at approximately 0.72 for any given PCR run or 0.98 per sample when run in triplicate [1 − (1–0.72) * (1–0.72) * (1–0.72)]. We also found estimated prevalence to be fairly consistent between years (approximately 28%; 95% confidence interval or CI: 25–32%) with limited support for sample year as an informative parameter (Table 2). Given the low prevalence of *Plasmodium* and *Haemoproteus* infections, we did not model the detection probability or prevalence of parasites of these genera among our sample of Emperor Geese nor did we assess potential detrimental fitness effects thereof.

**Table 2:** Model selection results examining the effect of sample year on *Leucocytozoon* detection (ρ_1_) and prevalence (ψ_1_) among Emperor Geese captured on the Yukon-Kuskokwim Delta, Alaska during 2006–2016 using an information theoretic approach. Fletcher's ĉ equaled 2.22 in this analysis. QAICc of the highest-ranking model was 586.94.

Model	ΔQAICc	Weight	Qdeviance	*K*
ρ_1_(.) ψ_1_(.)	0.00	0.95	90.00	2
ρ_1_(.)ψ_1_(Year)	6.05	0.05	75.55	12
ρ_1_(Year) ψ_1_(.)	10.42	0.01	79.91	12
ρ_1_(Year) ψ_1_(Year)	19.03	0.00	67.31	22

**Fig. 1:**
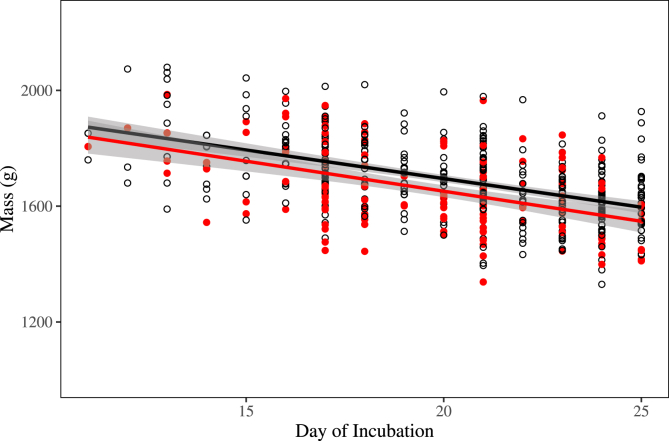
Mass of adult female nesting Emperor Geese per day of incubation for birds infected (red) and uninfected (black/white) with *Leucocytozoon* parasites using samples collected on the Yukon-Kuskokwim Delta, Alaska during 2006–2016. Trend lines indicate the predicted mass for an individual goose throughout the incubation period from day 11 based upon on the top supported model (Mass ~ Inc + Leu). (For interpretation of the references to colour in this figure legend, the reader is referred to the Web version of this article.)

Using regression analysis, we found evidence for a small effect of *Leucocytozoon* infection on the mass of incubating female geese (Table 3). More specifically, our results provided the strongest support for models explaining the mass of incubating female Emperor Geese as a function of incubation day and *Leucocytozoon* infection status, with weaker evidence for a year effect (Table 3). The most parsimonious model in our candidate set estimated the mass of incubating females infected with *Leucocytozoon* parasites to be approximately 43 g (95% CI: 20–67 g) less than uninfected females during the second half of incubation (days 11–25; Fig. 1). The rate of loss for both infected and uninfected females was estimated to be 20 g/day (95% CI: 17–23 g/day; Fig. 1).

**Table 3:** Model selection results for regression to assess mass of adult female nesting Emperor Geese as a function of day of incubation (Inc), *Leucocytozoon* infections status (Leu), and sample year. AICc of the highest-ranking model was 6123.95.

Model	ΔAICc	Weight	Likelihood	*K*
Mass ~ Inc + Leu	0.00	0.37	1.00	4
Mass ~ Inc + Leu + Year	0.01	0.37	1.00	14
Mass ~ Inc*Leu	1.96	0.14	0.38	5
Mass ~ Inc*Leu + Year	2.12	0.13	0.35	15
Mass ~ Inc + Year	10.13	0.00	0.01	13
Mass ~ Inc	11.35	0.00	0.00	3
Mass ~ Inc + Leu*Year	15.47	0.00	0.00	24
Mass ~ Inc*Year	27.12	0.00	0.00	23
Mass ~ Inc*Leu*Year	49.93	0.00	0.00	45

Using a multi-state hidden Markov framework, we did not find evidence for an effect of *Leucocytozoon* infection on survival of adult female Emperor Geese breeding on the Yukon-Kuskokwim Delta (Table 4). Specifically, our top-ranked model (AICc wi = 0.37) did not include parameters for survival differences between infected and uninfected geese (Table 4). Instead, our top model included parameters for resight probability based on infection status and state transition probabilities (e.g., switching from uninfected to infected and *vice versa*). Furthermore, survival rates from the best-supported model that incorporated parameters for infection status (ΔAICc = 1.93, AICc wi = 0.14) indicated that female geese infected with *Leucocytozoon* parasites (0.80; 95% CI: 0.72–0.86) had nearly identical survival rates to uninfected geese (0.79; 95% CI: 0.76–0.82). Annual survival rates of Emperor Geese were estimated to vary during our study period ranging from 0.66 (95% CI: 0.54–0.76) in 2007–2008 to 0.90 (95% CI: 0.76–0.96) in 2010–2011.

**Table 4:** Model selection results for influences on survival (ϕ), detection probabilities (ρ_2_), state transition probabilities (ψ_2_), probability of event at first capture (π), and probability of determining states (δ) of adult Emperor Geese captured on the Yukon-Kuskokwim Delta, Alaska during 2006–2016. Models include variables for blood parasite infection status of geese (state), and year. AICc of the highest-ranking model was 3032.97.

Model	ΔAICc	Weight	Likelihood	*K*	Deviance
ϕ(.) ρ_2_(state) ψ_2_(state) π(.) δ(.)	0.00	0.37	1.00	7	3359.48
ϕ(year) ρ_2_(state) ψ_2_(state) π(.) δ(.)	0.82	0.25	0.66	16	3341.89
ϕ(state) ρ_2_(state) ψ_2_(state) π(.) δ(.)	1.93	0.14	0.38	8	3359.38
ϕ(year) ρ_2_(state) ψ_2_(state) π(.) δ(state)	1.99	0.14	0.37	17	3341.00
ϕ(state) ρ_2_(state) ψ_2_(state) π(.) δ(state)	3.22	0.07	0.20	9	3358.64
ϕ(.) ρ_2_(.) ψ_2_(.) π(.) δ(.)	6.38	0.02	0.04	5	3369.91
ϕ(state) ρ_2_(state) ψ_2_(.) π(.) δ(.)	7.96	0.01	0.02	7	3367.44
ϕ(state) ρ_2_(.) ψ_2_(.) π(.) δ(.)	8.15	0.01	0.02	6	3369.66

**Fig. 2:**
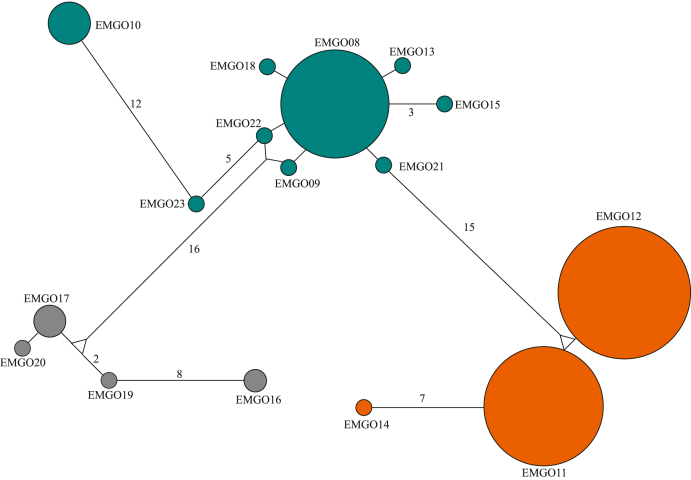
Minimum spanning network for haemosporidian mitochondrial DNA cytochrome *b* haplotypes identified from blood samples collected from Emperor Geese inhabiting the Yukon-Kuskokwim Delta, Alaska during 2006–2016. Circles are drawn proportional to the frequency at which haplotypes were detected. Shading represented the assignment of representative sequences for haplotypes to *L. simondi* clade A (teal), *L. simondi* clade B (orange), or other *Leucocytozoon* (grey) in phylogenetic analyses (see Results and Fig. 5). Lines are drawn proportional to genetic distance and are labeled per the number of mutations represented (except single nucleotide polymorphisms which are unlabeled). (For interpretation of the references to colour in this figure legend, the reader is referred to the Web version of this article.)

From the amplification and genetic sequencing of parasite mtDNA *cyt b* sequences, we identified 23 haplotypes among Emperor Geese infected with haemosporidian parasites assigned to the genera *Plasmodium* (n = 2), *Haemoproteus* (n = 5), and *Leucocytozoon* (n = 16; Table 5). Thirteen of these haplotypes were identical to those previously identified on public databases, including eight *Leucocytozoon* haplotypes (Table 5). All three parasite mtDNA *cyt b* haplotypes detected among at least ten individuals were of the genus *Leucocytozoon*, two of which (EMGO11, and EMGO12) were differentiated by a single nucleotide polymorphism (Fig. 2).

**Table 5:** Summary of haemosporidian parasite haplotypes identified through molecular screening of blood samples collected from Emperor Geese captured on the Yukon-Kuskokwim Delta, Alaska during 2006–2016.

Haplotype	inferred number of detections	novel or previously reported	identical or most closely related sequence on NCBI GenBank	identical or most closely related sequence on MalAvi database	Morphospecies of identical or most closely related sequence on GenBank/MalAvi databases	Host of identical or most closely related parasite sequence on GenBank/MalAvi databases
EMGO01	1	novel	KT193627	MELME/BT7	*P. circumflexum*	Passeriformes: Melospiza melodia
EMGO02	6	previously reported	KT193627	MELME/BT7	*P. circumflexum*	Passeriformes: *M. melodia*
EMGO03	1	previously reported	AF465591	CYGNUS01	*Haemoproteus* sp.	Anseriformes: *Cygnus columbianus*
EMGO04	1	novel	AF465591	CYGNUS01	*Haemoproteus* sp.	Anseriformes: *C.columbianus, Anas platyrhynchos*
EMGO05	1	previously reported	GQ141557	ANACRE01	*Haemoproteus* sp.	Anseriformes: *A. crecca*
EMGO06	1	previously reported	JQ314227	TUSW08	*Haemoproteus* sp.	Anseriformes: *C. columbianus*
EMGO07	1	previously reported	MG765392	CORCAU01	*Haemoproteus* sp.	Passeriformes: *Corvus caurinus*
EMGO08	44	previously reported	JQ314223	TUSW04	*Leucocytozoon* sp.	Anseriformes: *C. columbianus*
EMGO09	1	novel	JQ314222	TUSW03	*Leucocytozoon* sp.	Anseriformes: *C. columbianus*
EMGO10	7	previously reported	MG734973	SPEI05	*Leucocytozoon simondi*	Anseriformes: *Somateria fischeri*
EMGO11	55	previously reported	JQ314220	TUSW01	*Leucocytozoon* sp.	Anseriformes: *C. columbianus*
EMGO12	69	previously reported	JQ314221	TUSW02	*Leucocytozoon* sp.	Anseriformes: *C. columbianus*
EMGO13	1	novel	JQ314223	TUSW04	*Leucocytozoon* sp.	Anseriformes: *Cygnus columbianus*
EMGO14	1	novel	AB741512/AB741516	ANSFAB01	*Leucocytozoon* sp.	Anseriformes: *Anser fabalis*
EMGO15	1	previously reported	JQ314224	TUSW05	*Leucocytozoon* sp.	Anseriformes: *C. columbianus*
EMGO16	2	previously reported	KU842391	COBRA02	*Leucocytozoon* sp.	Passeriformes: *Corvus* sp.
EMGO17	4	previously reported	KR052961	COLBF29	*Leucocytozoon* sp.	Diptera: *Simulium silvestre*
EMGO18	1	novel	JQ314223	TUSW04	*Leucocytozoon* sp.	Anseriformes: *C. columbianus*
EMGO19	1	novel	KR052961	COLBF29	*Leucocytozoon* sp.	Diptera: *S. silvestre*
EMGO20	1	novel	KR052961	COLBF29	*Leucocytozoon* sp.	Diptera: *S. silvestre*
EMGO21	1	previously reported	KU363719	BWTE29	*Leucocytozoon* sp.	Anseriformes: *A. discors*
EMGO22	1	novel	JQ314222	TUSW03	*Leucocytozoon* sp.	Anseriformes: *C. columbianus*
EMGO23	1	novel	JQ314222	TUSW03	*Leucocytozoon* sp.	Anseriformes: *C. columbianus*

**Fig. 3:**
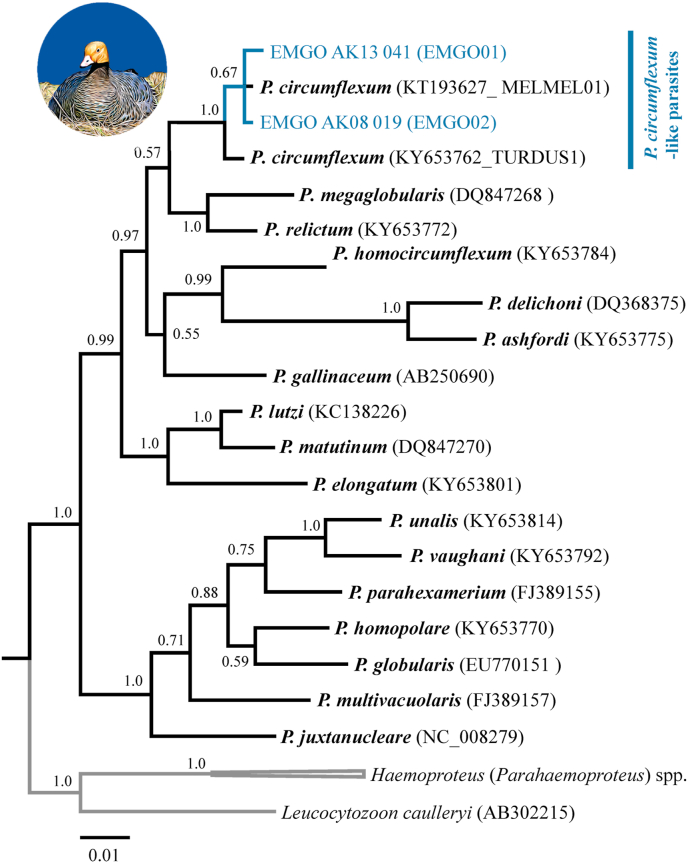
Phylogenetic tree depicting inferred genetic relationships among *Plasmodium* mitochondrial DNA cytochrome *b* haplotypes identified from blood samples collected from Emperor Geese inhabiting the Yukon-Kuskokwim Delta, Alaska during 2006–2016 and those previously reported for closely related haemosporidian morphospecies on the National Center for Biotechnology Information GenBank and Malavi databases (accession IDs in parentheses).

**Fig. 4:**
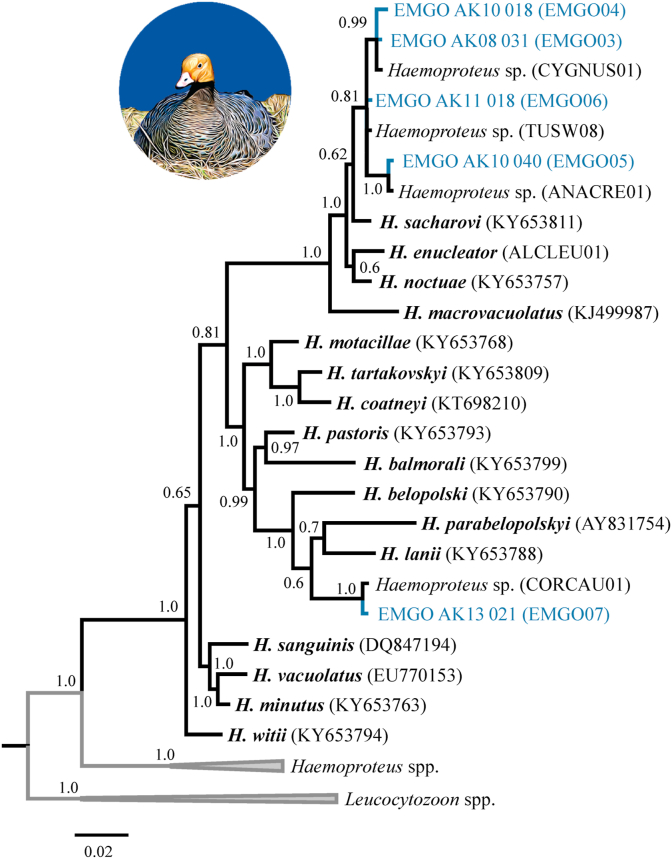
Phylogenetic tree depicting inferred genetic relationships among *Haemoproteus* mitochondrial DNA cytochrome *b* haplotypes identified from blood samples collected from Emperor Geese inhabiting the Yukon-Kuskokwim Delta, Alaska during 2006–2016 and those previously reported for closely related haemosporidian morphospecies on the National Center for Biotechnology Information GenBank and Malavi databases (accession IDs in parentheses).

**Fig. 5:**
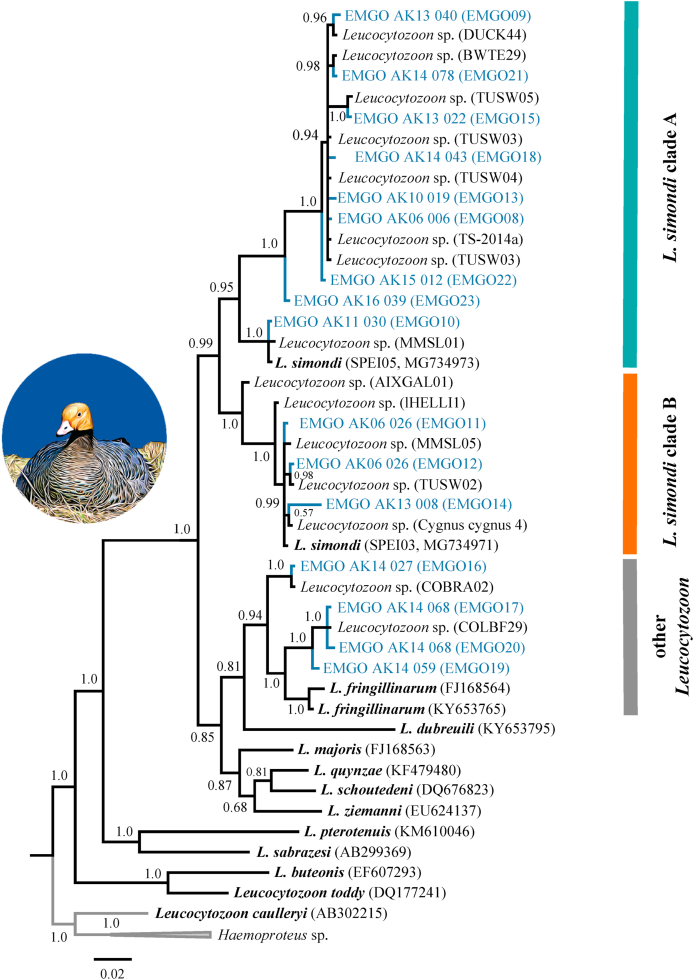
Phylogenetic tree depicting inferred genetic relationships among *Leucocytozoon* mitochondrial DNA cytochrome *b* haplotypes identified from blood samples collected from Emperor Geese inhabiting the Yukon-Kuskokwim Delta, Alaska during 2006–2016 and those previously reported for closely related haemosporidian morphospecies on the National Center for Biotechnology Information GenBank and Malavi databases (accession IDs in parentheses). Bars to the right of tree represent the assignment of sequences to *L. simondi* clade A (teal), *L. simondi* clade B (orange), or other *Leucocytozoon*. (For interpretation of the references to colour in this figure legend, the reader is referred to the Web version of this article.)

In our first phylogenetic analysis, both *Plasmodium* mtDNA *cyt b* haplotypes found in Emperor Goose blood samples formed a monophyletic clade with sequences previously reported for *Plasmodium circumflexum* (posterior probability 1.0), though the topology with a sister clade including sequences for *Plasmodium relictum* and *Plasmodium megaglobularis* was not conclusively resolved (posterior probability 0.57; Fig. 3). In our second phylogenetic analysis involving *Haemoproteus* mtDNA *cyt b* haplotypes, four parasite sequences from Emperor Goose formed a moderately supported clade (posterior probability 0.62) with haplotypes previously reported for *Haemoproteus sacharovi* (from doves) and unidentified *Haemoproteus* morphospecies detected in waterfowl (Fig. 4). A fifth *Haemoproteus* mtDNA *cyt b* haplotype from a single goose sample was nested within a strongly supported clade (posterior probability 1.0) with haplotypes previously reported for *Haemoproteus belopolski*, *Haemoproteus parabelopolskyi*, *Haemoproteus lanii*, and an unidentified *Haemoproteus* morphospecies (Fig. 4). In our final phylogenetic analysis, we found 16 representative *Leucocytozoon* mtDNA *cyt b* haplotypes sequences to cluster into three strongly supported major clades (posterior probabilities >0.94) with those previously reported on public databases (Fig. 5). Nine *Leucocytozoon* parasite mtDNA sequences from Emperor Goose blood samples formed a clade with a sequence previously identified for *L. simondi* as well as numerous other sequences previously detected in waterfowl samples from unidentified *Leucocytozoon* morphospecies. We designated this group of sequences as ‘*L. simondi* clade A’ (Fig. 5). Four additional *Leucocytozoon* parasite mtDNA sequences identified in goose blood samples were part of a sister clade with another sequence previously identified for *L. simondi* and several other sequences previously detected in waterfowl samples from unidentified *Leucocytozoon* morphospecies. We designated this group as ‘*L. simondi* clade B’ (Fig. 5). The four remaining *Leucocytozoon* parasite mtDNA sequences from Emperor Goose blood samples were part of a third clade with sequences previously reported for *L. fringillinarum* and an unidentified *Leucocytozoon* morphospecies (Fig. 5).

**Fig. 6:**
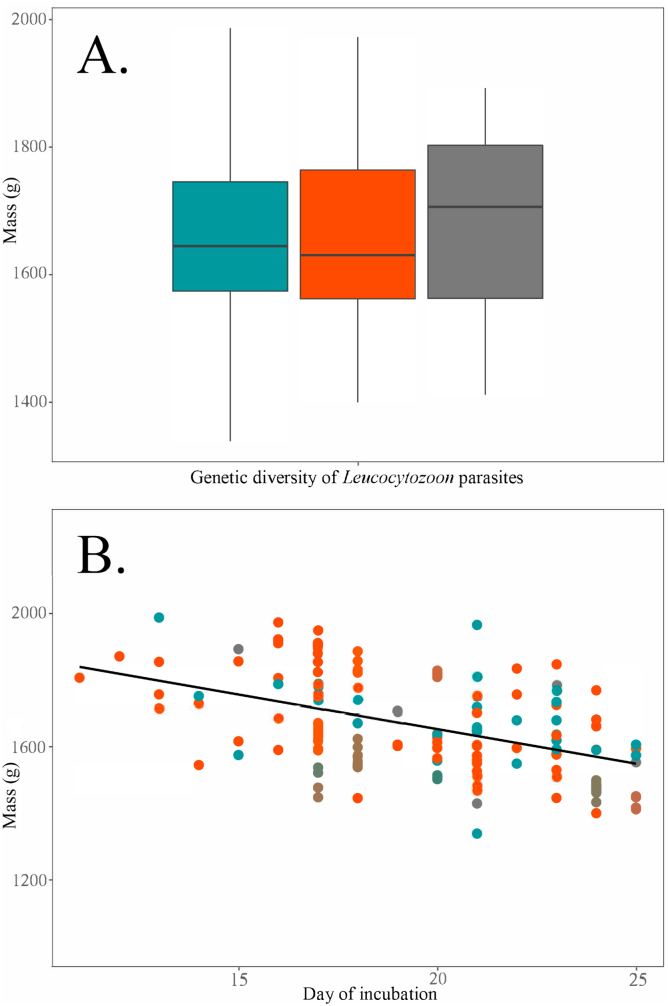
Comparison of mass measures for incubating adult female Emperor Geese infected with *Leucocytozoon* parasites genetically characterized in this study *L. simondi* clade A (blue), *L. simondi* clade B (red), or other/mixed *Leucocytozoon* (grey; see Materials and methods) using boxplots (Panel A) and plotted by incubation day (Panel B). The trendline in panel B is depicts predicted mass given the day of incubation and positive *Leucocytozoon* infection status from our top-ranking regression model (see Results). (For interpretation of the references to colour in this figure legend, the reader is referred to the Web version of this article.)

In a *post hoc* analysis to explore potential associations between genetic lineages of parasites and possible detrimental fitness effects, we did not find evidence that specific groups of phylogenetically related *Leucocytozoon* parasite haplotypes were consistently associated with differential mass measures among adult female nesting Emperor Geese (Fig. 6). That is, exploratory analyses did not support a difference in mass as a function of incubation day among adult females infected with *Leucocytozoon* parasites genetically characterized in this study as *L. simondi* clade A (F_3,144_ = 15.65) as compared to those infected with *L. simondi* clade B (p = 0.40) or other/mixed *Leucocytozoon* parasites (p = 0.39), nor between birds infected with *L. simondi* clade B parasites as compared to birds harboring parasites characterized as other/mixed *Leucocytozoon* (p = 0.18; Fig. 6).

## 4 Discussion

In this study, we found Emperor Geese inhabiting the Yukon-Kuskokwim Delta of Alaska to be infected with *Leucocytozoon* parasites in each of 11 sample years with an annual prevalence of approximately 28%. These results are consistent with the microscopic identification of *Leucocytozoon simondi* in an adult Emperor Goose sampled at this same location in 1996 (Hollmén et al., 1998) as well as two prior molecular assessments estimating the prevalence of *Leucocytozoon* parasites among adult Emperor Geese at this location to be approximately 20–27% (Ramey et al., 2014; Buchheit et al., 2021a). Furthermore, geese harbored phylogenetically diverse *Leucocytozoon* parasites, consistent with previously reports for parasites infecting other North American waterfowl species and suggestive of cryptic parasite speciation (Reeves et al., 2015; Pacheco et al., 2018; Pacheco and Escalante 2020). Additionally, we detected few infections with *Haemoproteus* parasites and even fewer infections with *Plasmodium* parasites. These findings are consistent with the infrequent molecular detection of *Haemoproteus* and *Plasmodium* parasites in Emperor Geese from the Yukon-Kuskokwim Delta in prior studies (Ramey et al., 2014; Buchheit et al., 2021a). Similar relative prevalence for parasites of these genera, including a higher prevalence of *Leucocytozoon* as compared to *Haemoproteus* and *Plasmodium* parasites, has been reported in Black Brant (*Branta bernicla nigricans*), Northern Pintails (*Anas acuta*), and Spectacled Eiders (*Somateria fischeri*) sampled on the Yukon-Kuskokwim Delta (Ramey et al., 2014, Ramey et al., 2015; Reed et al., 2018), which might be related to differences among these parasite genera with regard to minimum temperatures required for parasite development (Valkiunas 2004). We incorporated an occupancy modeling approach in the current study to account for imperfect sensitivity of molecular methods to detect haemosporidian parasites and better estimate prevalence, which achieved near-perfect detection probability (0.98) by incorporating triplicate PCR runs. Given the consistency of our findings with previously published work, including our relatively high estimated detection probability, we believe our results provide robust estimates of haemosporidian prevalence for Emperor Geese inhabiting the Yukon-Kuskokwim Delta.

Using a combination of regression analysis and multi-state hidden Markov modeling, we found negligible support for potential fitness consequences of *Leucocytozoon* infection among adult breeding Emperor Geese. Though our results provided evidence for an association between reduced mass and *Leucocytozoon* infection among incubating females during the second half of incubation, the effect was relatively small (approximately 2% of total mass) and appeared unlikely to lead to detrimental fitness consequences as inferred from the lack of support for an effect of *Leucocytozoon* infection on annual survival. Furthermore, our genetic analyses indicated that the most common parasite lineages detected in adult female nesting Emperor Geese may also be common in waterfowl and other birds sampled elsewhere. To our knowledge, these parasite lineages have not previously been associated with widespread clinical disease in birds. Finally, our *post-hoc* analyses did not provide evidence for specific clades of *Leucocytozoon* parasites as being associated with comparatively lower mass among infected nesting adult female Emperor Geese. Therefore, our collective results provide negligible evidence for *Leucocytozoon* parasites as causing detrimental effects to adult breeding female Emperor Geese on the Yukon-Kuskokwim Delta, and indicate that these parasites are unlikely to represent a contemporary disease threat to the population health of this species.

Though our results did not provide clear evidence for fitness consequences of haemosporidian infections among nesting adult female Emperor Geese, we acknowledge limitations of our study design that precluded rigorous inference pertaining to potential effects of parasites at the individual level, to other age/sex cohorts, and birds infected under adverse ecological conditions (e.g., during extended periods of nutritional stress or prolonged exposure to extreme weather events). For example, as our molecular approach did not evaluate the parasitemia of haemosporidian infections or distinguish between pre-erythrocytic or patent infections, it is plausible that we failed to recognize important fitness consequences of birds suffering from high intensity, acute parasite infections. Similarly, our investigation did not assess the potential fitness consequences of relatively rare parasite genera and haplotypes. Our sample was almost entirely of birds of the same breeding status and age/sex cohort and we did not stratify our analyses based upon ecological conditions encountered by birds during the breeding season. Therefore, the current evaluation should not be considered exhaustive and future assessments of potential effects of haemosporidian parasites may be warranted, particularly if mortality events occur that are suspected of being the result of avian malaria or Leucocytozoonosis, or if observations are made indicating increases in occurrence of *Haemoproteus* or *Plasmodium* parasites among birds inhabiting the Yukon-Kuskokwim Delta.

## 5 Conclusions

Using molecular analysis, we found evidence that Emperor Geese inhabiting the Yukon-Kuskokwim Delta, Alaska are consistently infected with genetically diverse *Leucocytozoon* parasites at a prevalence of approximately 28%. Through the application of modeling approaches, we found evidence for a small effect of *Leucocytozoon* infection on the mass of incubating adult female Emperor Geese; however, we did not find support for an effect of *Leucocytozoon* infection on survival. Furthermore, we did not find evidence that parasites assigned to two genetically diverse clades were associated with differential mass measures among nesting adult female Emperor Geese. Collectively, our results provide negligible evidence for *Leucocytozoon* parasites as causing detrimental effects to adult female Emperor Geese breeding on the Yukon-Kuskokwim Delta.

## Declaration of competing interest

The authors declare that they have no conflicts of interest.

## References

[bib1] Altschul S.F., Madden T.L., Schäffer A.A., Zhang J., Zhang Z., Miller W., Lipman D.J. (1997). Gapped BLAST and PSI-BLAST: a new generation of protein database search programs. Nucleic Acids Res..

[bib46] Bandelt H.J., Forster P., Röhl A. (1999). Median-joining networks for inferring intraspecific phylogenies. Mol. Biol. Evol..

[bib44] Bensch S., Hellgren O., Perz-Tris J. (2009). MalAvi: a public database of malaria parasites and related haemosporidians in avian hosts based on mitochondrial cytochrome b lineages. Mol. Ecol. Res..

[bib45] Benson D.A., Cavanaugh M., Clark K., Karsch-Mizrachi I., Lipman D.J., Ostell J., Sayers E.W. (2012). GenBank. Nucleic Acids Res..

[bib2] BirdLife International (2016). *Anser canagicus*. The IUCN Red List of Threatened Species 2016: e.T22679919A92834737.

[bib3] Buchheit R.M., Schmutz J.A., Reed J.A., Uher-Koch B., Ramey A.M. (2021). Assessment of variation in the detection and prevalence of blood parasites among sympatrically breeding geese in western Alaska. J. Wildl. Dis..

[bib4] Buchheit R.M., Uher-Koch B.D., Schmutz J.A., Reed J.A., Pacheco M.A., Escalante A.A., Ramey A.M. (2021).

[bib5] Burnham K.P., Anderson D.R. (2002). Model Selection and Multimodel Inference: A Practical Information-Theoretic Approach.

[bib6] Conn P.B., Cooch E.G. (2009). Multistate capture–recapture analysis under imperfect state observation: an application to disease models. J. Appl. Ecol..

[bib7] Desser S.S., Ryckman A.K. (1976). The development and pathogenesis of *Leucocytozoon simondi* in Canada and domestic geese in Algonquin Park, Ontario. Can. J. Zool..

[bib8] Dimitrov K.M., Ramey A.M., Qiu X., Bahl J., Afonso C.L. (2016). Temporal, geographic, and host distribution of avian paramyxovirus 1 (Newcastle disease virus). Infect. Genet. Evol..

[bib9] Fischer J.B., Williams A.R., Stehn R.A. (2017).

[bib10] Fleskes J.P., Ramey A.M., Reeves A.B., Yee J.L. (2017). Body mass, wing length, and condition of wintering ducks relative to hematozoa infection. J. Fish Wildlife Manag..

[bib11] Gouy M., Guindon S., Gascuel O. (2010). SeaView version 4: a multiplatform graphical user interface for sequence alignment and phylogenetic tree building. Mol. Biol. Evol..

[bib12] Hellgren O., Waldenström J., Bensch S. (2004). A new PCR assay for simultaneous studies of *Leucocytozoon, Plasmodium*, and *Haemoproteus* from avian blood. J. Parasitol..

[bib13] Herman C.M., Barrow J.H., Tarshis I.B. (1975). Leucocytozoonosis in Canada geese at the seney national Wildlife refuge. J. Wildl. Dis..

[bib14] Hollmén T.E., Franson J.C., Creekmore L.H., Schmutz J.A., Fowler A.C. (1998). *Leucocytozoon simondi* in emperor geese from the yukon-kuskokwim Delta in Alaska. Condor.

[bib15] Kerr K.C., Stoeckle M.Y., Dove C.J., Weigt L.A., Francis C.M., Hebert P.D. (2007). Comprehensive DNA barcode coverage of North American birds. Mol. Ecol. Notes.

[bib16] Kumar S., Stecher G., Tamura K. (2016). MEGA7: molecular evolutionary genetics analysis version 7.0 for bigger datasets. Mol. Biol. Evol..

[bib17] Lee D.H., Bertran K., Kwon J.H., Swayne D.E. (2017). Evolution, global spread, and pathogenicity of highly pathogenic avian influenza H5Nx clade 2.3.4.4. J. Vet. Sci..

[bib18] Longmire J.L., Lewis A.K., Brown N.C., Buckingham J.M., Clark L.M., Jones M.D. (1988). Isolation and molecular characterization of a highly polymorphic centromeric tandem repeat in the family Falconidae. Genomics.

[bib19] Meixell B.W., Arnold T.W., Lindberg M.S., Smith M.M., Runstadler J.A., Ramey A.M. (2016). Detection, prevalence, and transmission of avian hematozoa in waterfowl at the Arctic/sub-Arctic interface: co-infections, viral interactions, and sources of variation. Parasites Vectors.

[bib20] Merrill L., Levengood J.M., England J.C., Osborn J.M., Hagy H.M. (2018). Blood parasite infection linked to condition of spring-migrating Lesser Scaup (*Aythya affinis*). Can. J. Zool..

[bib21] Miłek J., Blicharz-Domańska K. (2018). Coronaviruses in avian species–review with focus on epidemiology and diagnosis in wild birds. J. Vet. Res..

[bib22] Muradrasoli S., Wahlgren J., Waldenström J., Belák S., Blomberg J., Olsen B., Bálint, Á. (2010). Prevalence and phylogeny of coronaviruses in wild birds from the Bering Strait area (Beringia). PloS One.

[bib23] Pacheco M.A., Escalante A.A., Santiago-Alarcon D., Marzal A. (2020). Avian Malaria and Related Parasites in the Tropics: Ecology, Evolution and Systematics.

[bib24] Pacheco M.A., Matta N.E., Valkiunas G., Parker P.G., Mello B., Stanley C.E., Lentino M., Garcia-Amado M.A., Cranfield M., Pond S.L.K., Escalante A.A. (2018). Mode and rate of evolution of haemosporidian mitochondrial genomes: timing the radiation of avian parasites. Mol. Biol. Evol..

[bib25] Pacific Flyway Council (2016).

[bib26] R Core Team (2021).

[bib27] Ramey A.M., Ely C.R., Schmutz J.A., Pearce J.M., Heard D.J. (2012). Molecular detection of hematozoa infections in Tundra Swans relative to migration patterns and ecological conditions at breeding grounds. PloS One.

[bib28] Ramey A.M., Reeves A.B., Ogawa H., Ip H.S., Imai K., Bui V.N., Yamaguchi E., Silko N.Y., Afonso C.L. (2013). Genetic diversity and mutation of avian paramyxovirus serotype 1 (Newcastle disease virus) in wild birds and evidence for intercontinental spread. Arch. Virol..

[bib29] Ramey A.M., Reed J.A., Schmutz J.A., Fondell T.F., Meixell B.W., Hupp J.W., Ward D.H., Terenzi J., Ely C.R. (2014). Prevalence, transmission, and genetic diversity of blood parasites infecting tundra-nesting geese in Alaska. Can. J. Zool..

[bib30] Ramey A.M., Schmutz J.A., Reed J.A., Fujita G., Scotton B.D., Casler B., Fleskes J.P., Konishi K., Uchida K., Yabsley M.J. (2015). Evidence for intercontinental parasite exchange through molecular detection and characterization of haematozoa in Northern Pintails (*Anas acuta*) sampled throughout the North Pacific Basin. Int. J. Parasitol. Parasites Wildl..

[bib31] Ramey A.M., Uher‐Koch B.D., Reeves A.B., Schmutz J.A., Poulson R.L., Stallknecht D.E. (2019). Emperor Geese (*Anser canagicus*) are exposed to a diversity of influenza A viruses, are infected during the non‐breeding period and contribute to intercontinental viral dispersal. Transbound. Emerg. Dis..

[bib32] Reed J.A., Sexson M.G., Smith M.M., Schmutz J.A., Ramey A.M. (2018). Evidence for haemosporidian parasite infections in Spectacled Eiders (*Somateria fischeri*) sampled in Alaska, USA during the breeding season. J. Wildl. Dis..

[bib33] Reeves A.B., Pearce J.M., Ramey A.M., Ely C.R., Schmutz J.A., Flint P.L., Derksen D.V., Ip H.S., Trust K.A. (2013). Genomic analysis of avian influenza viruses from waterfowl in western Alaska, USA. J. Wildl. Dis..

[bib34] Reeves A.B., Smith M.M., Meixell B.W., Fleskes J.P., Ramey A.M. (2015). Genetic diversity and host specificity varies across three genera of blood parasites in ducks of the Pacific Americas flyway. PloS One.

[bib35] Reeves A.B., Hall J.S., Poulson R.L., Donnelly T., Stallknecht D.E., Ramey A.M. (2018). Influenza A virus recovery, diversity, and intercontinental exchange: a multi-year assessment of wild bird sampling at Izembek National Wildlife Refuge, Alaska. PloS One.

[bib36] Ronquist F., Huelsenbeck J.P. (2003). MrBayes 3: Bayesian phylogenetic inference under mixed models. Bioinformatics.

[bib37] Schmutz J.A., Petersen M.R., Rockwell R.F., Poole A.F. (2020). Birds of the World.

[bib38] Shutler D., Clark R.G., Rutherford S.T., Mullie A. (1999). Blood parasites, clutch volume, and condition of Gadwalls and Mallards. J. Avian Biol..

[bib39] Sorci G., Møller A.P. (1997). Comparative evidence for a positive correlation between haematozoan prevalence and mortality in waterfowl. J. Evol. Biol..

[bib40] Swayne D.E. (2009).

[bib41] Thompson S.C., Raveling D.G. (1987). Incubation behavior of Emperor Geese compared with other geese: interactions of predation, body size, and energetics. Auk.

[bib42] Valkiunas G. (2004).

[bib43] White G.C., Burnham K.P. (1999). Program MARK: survival estimation from populations of marked animals. Hous. Theor. Soc..

